# Exploring new horizons

**DOI:** 10.7554/eLife.23624

**Published:** 2017-01-04

**Authors:** Vineetha M Zacharia, Matthew F Traxler

**Affiliations:** 1 Department of Plant and Microbial Biology, University of California, Berkeley, Berkeley, United States; 1 Department of Plant and Microbial Biology, University of California, Berkeley, Berkeley, United Statesmtrax@berkeley.edu

**Keywords:** *Streptomyces*, volatile compound, alkaline, fungal interaction, trimethylamine, Other

## Abstract

*Streptomyces* bacteria employ a newly-discovered cell type, the "explorer" cell, to rapidly colonize new areas in the face of competition.

**Related research article** Jones SE, Ho L, Rees CA, Hill JE, Nodwell JR, Elliot MA. 2017. *Streptomyces* exploration is triggered by fungal interactions and volatile signals. *eLife* **6**:e21738. doi: 10.7554/eLife.21738

Historically, bacteria have been thought of as simple cells whose only aim is to replicate. However, research over the past two decades has revealed that many types of bacteria are able to develop into communities that contain several types of cells, with different cell types performing particular roles (Kuchina et al., 2011). These communities are of interest in scientific fields as diverse as petroleum engineering and bacterial pathogenesis.

*Streptomyces* were perhaps the first bacteria to be recognized as having a multicellular lifestyle (Waksman and Henrici, 1943). In fact, this lifestyle led to them being classified as fungi when they were first isolated from soil at the beginning of the last century (Hopwood, 2007). This case of mistaken identity stemmed from the fuzzy texture of *Streptomyces* colonies (see Figure 1A), which resembles many of the fungi we see growing on bread and other natural surfaces (Waksman, 1954).

**Figure 1.**
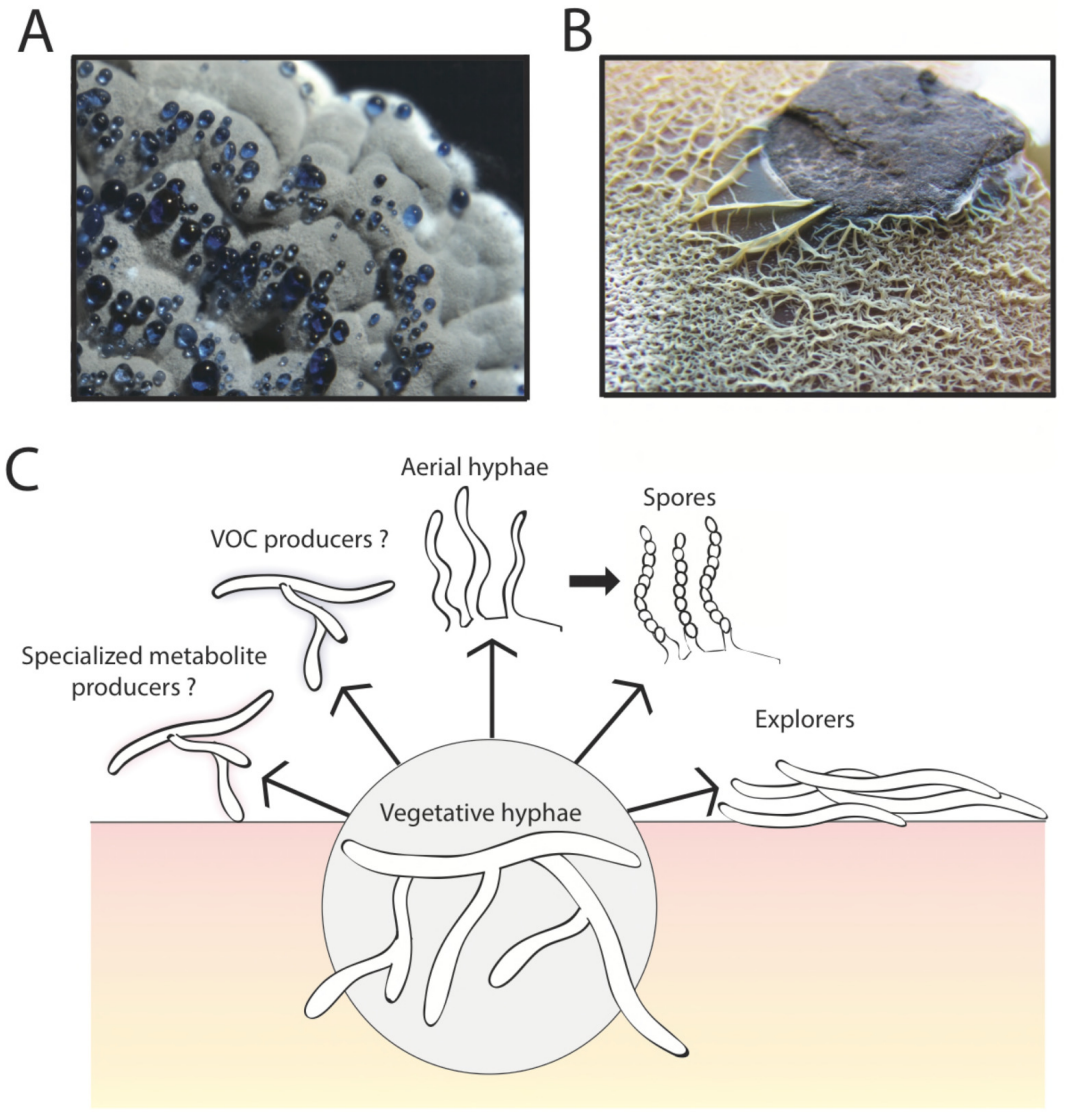
The multicellular lifestyle of *Streptomyces*. *Streptomyces* bacteria form colonies that contain several different types of specialized cells: vegetative hyphae, aerial hyphae, spores and the "explorer" cells discovered by Jones et al. (**A**) A *Streptomyces coelicolor* colony exhibiting aerial hyphae (white) and spores (gray). The blue droplets contain compounds that are naturally produced by *S. coelicolor* including antibiotics. (**B**) *Streptomyces venezuelae* explorer cells spreading on a rock. (**C**) In addition to these four types of cells, it is possible that *Streptomyces* colonies might contain other cell types that produce specialized metabolites, such as antibiotics, signaling molecules or volatile organic compounds (VOCs).

Image credits: panel A and C Vineetha Zacharia; panel B Jones et al. (2016)

The first stage in the life of a *Streptomyces* colony is the growth of so-called vegetative cells, which form networks of branched filaments that penetrate the surfaces of food sources. The fuzzy appearance of *Streptomyces* colonies is the result of the vegetative cells producing another type of cell called aerial hyphae that grow upwards into the air (McCormick and Flärdh, 2012; Flärdh and Buttner 2009). Subsequently, cells of a third type (spores) form long chains on the ends of these aerial hyphae. These spores are resistant to drying out and likely allow *Streptomyces* to passively spread to new environments through the action of water or air movement (McCormick and Flärdh, 2012). Now, in eLife, Marie Elliot at McMaster University and colleagues – including Stephanie Jones as first author – report a new form of growth in *Streptomyces* termed “exploratory growth” (Jones et al., 2016).

In the initial experiments, Jones et al. – who are based at McMaster University, the University of Toronto and Dartmouth College – grew *Streptomyces venezuelae* bacteria alone, or close to a yeast called *Saccharomyces cerevisiae,* on solid agar for two weeks. During this time, the bacteria grown alone formed a normal sized colony typical of *Streptomyces*. However, in the presence of the yeast, the *S. venezuelae* colonies expanded rapidly and colonized the entire surface of the growth dish, engulfing the nearby yeast colony. In subsequent experiments, the cells produced during exploratory growth (dubbed “explorer” cells) showed the ability to spread over abiotic surfaces including rocks (Figure 1B) and polystyrene barriers. Scanning electron microscopy revealed that, unlike vegetative cells, these explorer cells did not form branches and more closely resembled simple aerial hyphae.

Previous studies have identified many genes that regulate the development of *Streptomyces* colonies including the *bld* genes, which are involved in the formation of aerial hyphae, and the *whi* genes, which are required to make spores (McCormick and Flärdh, 2012). Jones et al. found that neither of these sets of genes are required for exploratory growth of *S. venezuelae* in the presence of the yeast. This suggests that the explorer cell type is distinct from the previously known developmental pathways in *Streptomyces*. Furthermore, Jones et al. found that multiple *Streptomyces* species were capable of exploratory growth and that various fungal microbes had the ability to trigger this behavior.

Further experiments using libraries of mutant yeast indicated that glucose and pH may be involved in triggering the formation of explorer cells. Jones et al. demonstrated that *Streptomyces* displays exploratory growth in response to shortages of glucose (caused by the presence of the yeast) and to an increased pH in the surrounding environment. The bacteria trigger this pH change themselves by releasing a volatile organic compound called trimethylamine, which is able to stimulate exploratory growth in *Streptomyces* over considerable distances. Trimethylamine also inhibits the growth of other bacteria that might compete with *S. venezuelae* in natural environments.

The work of Jones et al. opens up the possibility that there may be additional types of specialized cells within *Streptomyces* colonies. *Streptomyces* are important for medicine because they produce many different chemical compounds, including antibiotics and immunosuppressant drugs, and one might imagine that specific groups of cells within a colony are responsible for making these compounds (Figure 1C). Perhaps other cell types might be dedicated to directing the activities of different cells within the colony (as happens in other bacteria with multicellular lifestyles; Lopez et al., 2009; Baker, 1994), perhaps by producing trimethylamine or other volatile organic compounds.

For decades, researchers have described *Streptomyces* colonies in terms of vegetative cells, aerial hyphae and spores. The explorer cells identified by Jones et al. offer *Streptomyces* an alternative means of escape from their normal life cycle and local environment in the face of competition. This makes intuitive sense, given that *Streptomyces* lack the ability to move (“motility”) in the traditional sense (for example, by swimming, gliding or twitching). Taken together, the work of Jones et al. demonstrates a surprisingly dynamic strategy in which a ‘non-motile’ bacterium can use cues from other microbes, long-range signaling, and multicellularity to make a graceful exit when times get tough.
